# Tensile Property of Irradiated LT21 Aluminum Alloy Sampled from Decommissioned Irradiation Channel of Heavy Water Research Reactor

**DOI:** 10.3390/ma16020544

**Published:** 2023-01-05

**Authors:** Wanhuan Yang, Jin Qian, Weihua Zhong, Guangsheng Ning, Shunmi Peng, Wen Yang

**Affiliations:** Reactor Engineering Technology Research Institute, China Institute of Atomic Energy, Beijing 102413, China

**Keywords:** aluminum alloy, neutron irradiation, tensile property, irradiation embrittlement, transmutation Si

## Abstract

LT21 a type of aluminum alloy used for the irradiation channel of the first heavy water research reactor (HWRR) in China. Studying the mechanical property of irradiated LT21 aluminum under actual service conditions is essential for evaluating its application property. In this paper, tensile specimens of irradiated LT21 were manufactured from the decommissioned irradiation channel of an HWRR; then, tensile tests were carried out, and then the fracture surfaces were observed. The effect of neutron irradiation on tensile behavior and the failure mechanism was analyzed by comparing the result of irradiated and unirradiated LT21 specimens. The results show that, with the thermal neutron flux increasing to 2.38 × 10^22^ n/cm^2^, the YS gradually increased from the initial 158 MPa to 251 MPa, the UTS increased from 262 MPa to 321 MPa, and the elongation decreased from 28.8% to about 14.3%; the brittle fracture of the LT21 specimen appeared after irradiation, and the proportion of brittle fracture increased as the neutron fluence increased; the nanophase structures, with a size of less than 50 nm, were precipitated in the LT21 aluminum alloy after neutron irradiation. Transmutation Si is presumed to be the main cause of the radiation effect mechanism of LT21.

## 1. Introduction

Aluminum and its alloy have a low melting point. Thus, they have better resistance to the irradiation effect than other metals at low operating temperatures [1,2,3]. Aluminum is widely used in structural components of research reactors, including the High Flux Isotope Reactor (HFIR), the Savannah River Laboratory (SRL), and the Oak Ridge Research Reactor (ORRR) [4,5,6,7]. Munitz et al. [8] studied the effects of neutron irradiation on the mechanical properties and fracture morphology of Al-6063 using scanning electron microscopy (SEM), transmission electron microscopy (TEM), and the tensile tests method. Their results show that with the increase in neutron fluence, the uniform elongation and ultimate tensile strength increase, the intergranular fracture area increases, in addition to the fracture mechanism changes from ductile transgranular shear fracture to the combination of transgranular shear and intergranular dimple fracture. Gussev et al. [9] studied the effect of neutron irradiation on the mechanical properties and fracture behavior of Al-6061 alloy manufactured using ultrasonic additive. Their results show that the specimens exhibit noticeable hardening, and the ductility of the specimens decreases after radiation. The fracture mechanism of the specimen is mainly a ductile-type fracture with many dimples on the fracture surface.

LT21 is an Aluminum alloy used in the irradiation channel of the first HWRR in China. It is significant to study the mechanical properties of irradiated aluminum alloy under experimental conditions to evaluate its application performance [10,11,12]. In this paper, the tensile specimens of LT21 were manufactured using an irradiated channel decommissioned by the HWRR. By comparing the experimental results of irradiated and non-irradiated LT21 specimens, the effect of neutron irradiation on the tensile properties of LT21 aluminum alloy was discussed, and the failure mechanism of LT21 aluminum alloy before and after irradiation was analyzed.

## 2. Material and Experiment

### 2.1. Material

**Figure 1:**
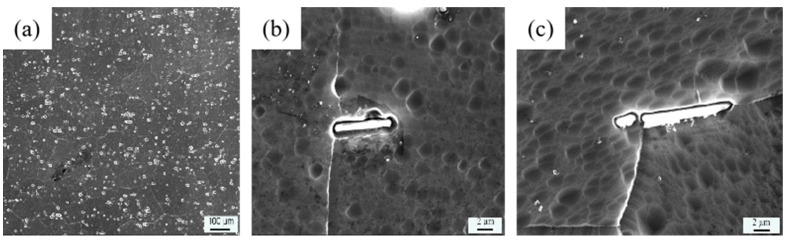
Metallograph of LT21 aluminum alloy: (**a**) Al matrix; (**b**) precipitate in the matrix; (**c**) precipitate at the grain boundary.

The test materials are irradiated and unirradiated LT21 aluminum from the decommissioned irradiation channel of HWRR. The dimension of the irradiation channel is 53 (outside diameter) × 1.5 (thickness) mm^2^. The main chemical compositions of LT21 aluminum alloy are as shown in Table 1. Figure 1 shows the metallograph of LT21 aluminum alloy. The average grain size is about 108 µm according to the direct measurement result of metallograph, and the primary phase is Al with BCC structure. The rod-like precipitates, which are composed of Mg and Si, are observed either in the Al matrix or at the grain boundary; the length is about 2.8~3.5 µm, and the width is about 1 µm; the coarse rod-shaped precipitates can be found in some place of matrix, with the length at about 10 µm.

The irradiated specimens were sampled from 3 irradiation channels with different service times. The operation parameters are as follows: effective full power day is 801–3020 days, the fluence range of thermal neutron (<0.625 eV) is 5.4 × 10^20^~2.38 × 10^22^ n/cm^2^, while the fluence range of fast neutron (>0.1 MeV) is 4.57 × 10^17^~2.37 × 10^19^ n/cm^2^. The thermal to fast neutrons ratio (TFR) is about 1000. The operation temperature is about 70 °C (343 K).

### 2.2. Experiment

**Figure 2:**
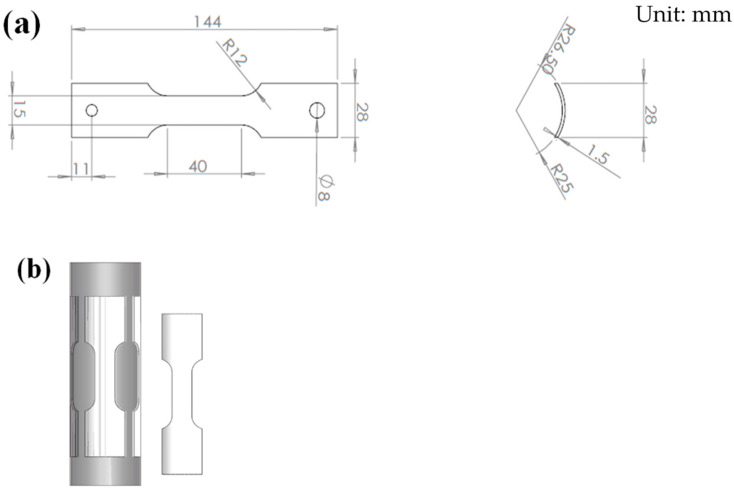
Tensile specimen size and sampling schematic diagram of the irradiation channel tube: (**a**) tensile specimen size; (**b**) sampling schematic diagram.

**Figure 3:**
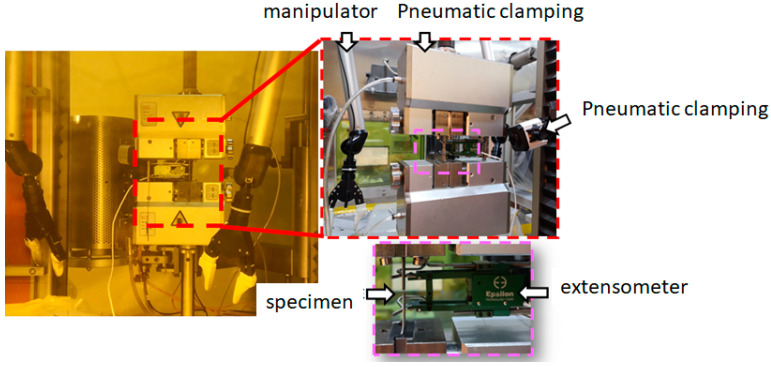
Tensile test machine in hot cell.

Tensile specimens were machined by using a vertical machining center in the hot cell. The dimension of the specimen and sampling schematic is shown in Figure 2. As shown in Figure 3, tensile tests were carried out on a Zwick/Roell Z100 testing machine in a hot cell at ambient temperature, with a test speed of 0.00025 s^−1^. The extensometer used for tensile tests is Epsilon SN E91695, with an accuracy level of 0.1, and the gage length is 25 mm.

After the test, the fracture surfaces of both irradiated and unirradiated specimens were observed by KYKY-EM6900 SEM in the hot cell. The microstructures of both irradiated and unirradiated specimens were observed by JOEL 2100F TEM. The TEM sample preparation process is as follows: the 10 × 10 mm^2^ sized sample was cut from both irradiated and unirradiated LT21 aluminum alloy tube; then, mechanical pre-thinning was carried out in the hot cell until the thickness of the sample became 100~150 nm, and finally, the Φ3 mm TEM samples were prepared by punch and Twin-jet electropolishing successively. The TEM was operated at 200 kV accelerating voltage. And the microstructure of the LT21 aluminum alloy matrix was observed by bright field image. Under the condition of matrix deviation diffraction, the second-phase particles were imaged.

## 3. Results

### 3.1. Tensile Test Results

**Figure 4:**
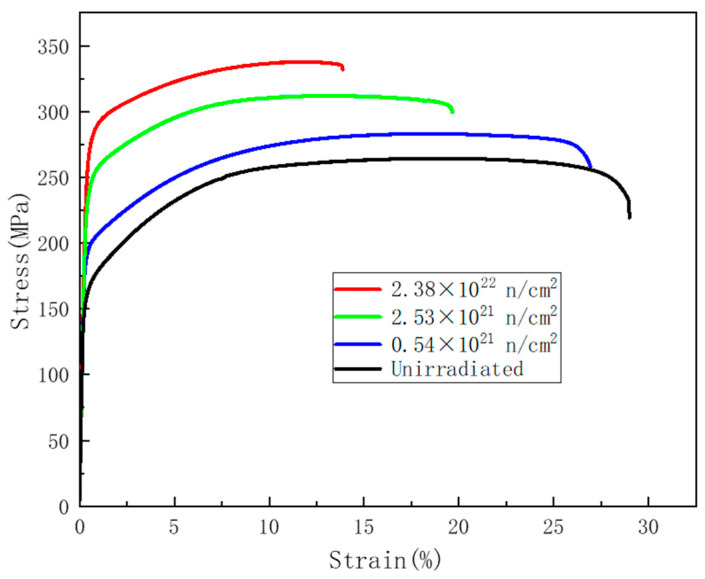
Stress–strain curve of the tensile specimen with different irradiation fluence.

Figure 4 shows the stress–strain curve of the LT21 aluminum tensile specimen with different irradiation fluence. There are three deformation stages that exist in both irradiated and unirradiated specimens: elastic deformation, plastic deformation, and instability deformation [4]. As the irradiation fluence increased, the strength of LT21 increased while the total elongation rate decreased.

**Figure 5:**
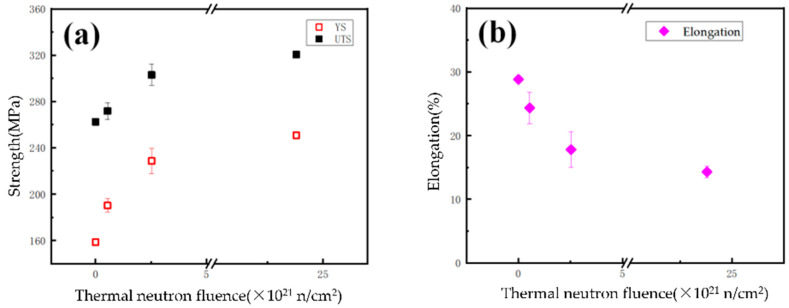
The relationship between the tensile properties of LT21 and the thermal neutron fluence: (**a**) strength vs thermal neutron fluence; (**b**) elongation vs thermal neutron fluence.

Figure 5 shows the relationship between the tensile properties of LT21 and the thermal neutron fluence. The tensile properties change exponentially with the fluence increase. As the thermal neutron fluence increased up to 2.38 × 10^22^ n/cm^2^, the yield stress (YS) increased gradually from the initial 158 MPa to 251 MPa, and the ultimate tensile stress (UTS) increased from 262 MPa to 321 MPa, while the elongation decreased from 28.8% to about 14.3%. The above test data indicated that, to the LT21, both the YS and UTS increased, while elongation decreased after neutron irradiation. Additionally, this phenomenon obviously proved that irradiation hardening and embrittlement occurred.

### 3.2. Fracture Surface Morphology Observation

**Figure 6:**
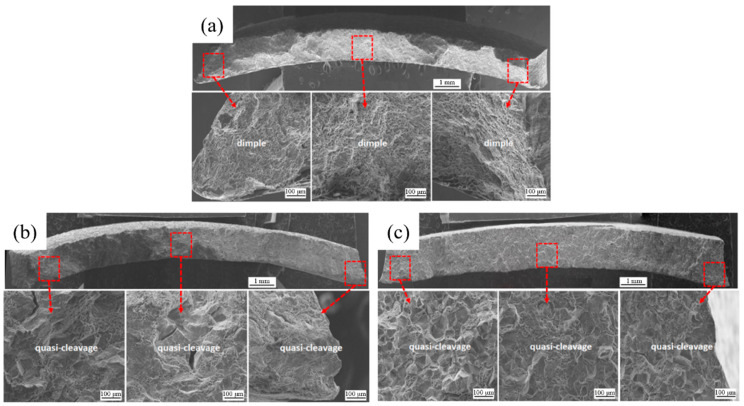
Fracture surface morphology of tensile specimen with different irradiation fluence: (**a**) unirradiated specimen; (**b**) irradiation fluence of 0.54 × 10^21^ n/cm^2^; (**c**) irradiation fluence of 2.38 × 10^22^ n/cm^2^.

Figure 6 shows the overall fracture morphology of tensile specimens with different irradiation fluence. The fracture characteristics of the edge and center are consistent with each other. The fracture surface of the unirradiated specimen shows dimple fracture characteristics, while the irradiated ones show quasi-cleavage characteristics.

**Figure 7:**
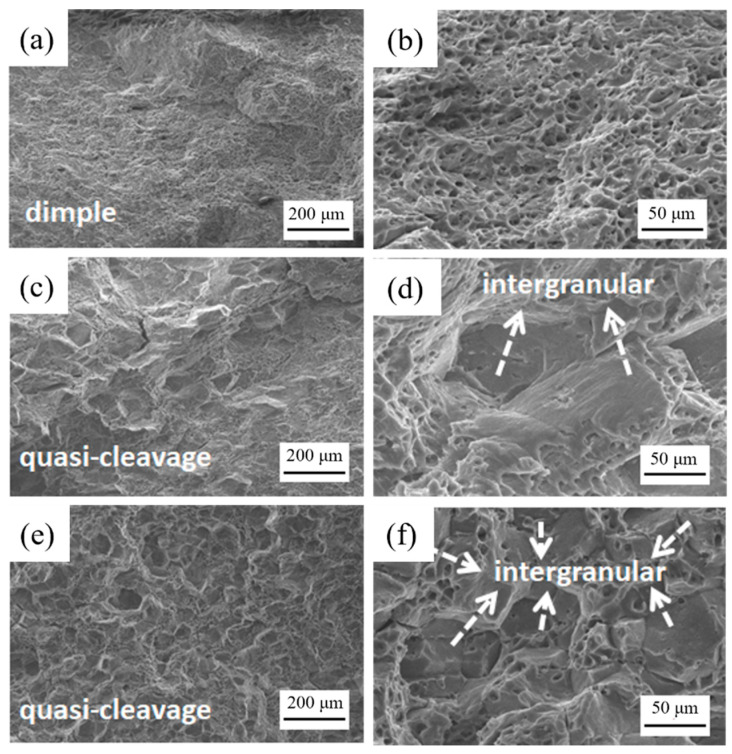
Local characteristics of tensile fracture of samples with different irradiation fluence: (**a**,**b**) unirradiated specimen; (**c**,**d**) thermal neutron fluence of 0.54 × 10^21^ n/cm^2^; (**e**,**f**) thermal neutron fluence of 2.38 × 10^22^ n/cm^2^.

Figure 7 shows the local morphology of the fracture surface. For the unirradiated specimen, the fracture surface morphology is mainly in dimple character, while for the low-fluence of the 0.54 × 10^21^ n/cm^2^ specimen, there are intergranular morphologies mixed with dimples, and it shows a quasi-cleavage character. The fracture surface of the high-fluence tensile specimen with the thermal fluence of 2.38 × 10^22^ n/cm^2^ is similar to that of 0.54 × 10^21^ n/cm^2^, which is in quasi-cleavage morphology [13], but the proportion of intergranular cleavage was more than that of lower irradiation fluence. This phenomenon indicates that, after irradiation, the brittle fracture of the LT21 specimen appeared, and the proportion of brittle fracture increased as the fluence increased. Therefore, it can be concluded that LT21 aluminum alloy was embrittled after neutron irradiation; however, the toughness remained on the specimen with a thermal neutron fluence of 2.38 × 10^22^ n/cm^2^.

**Figure 8:**
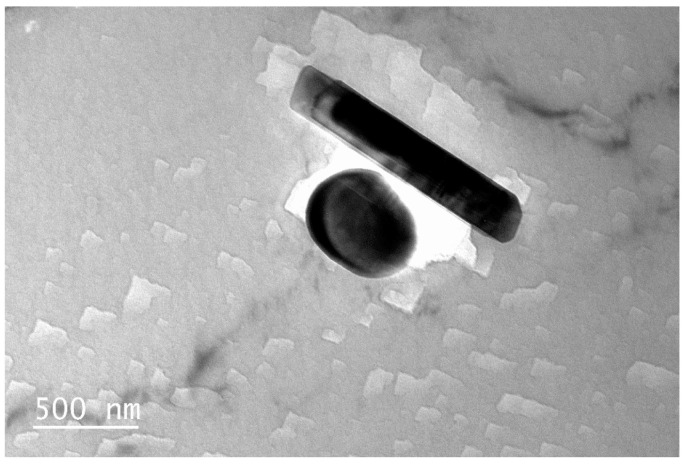
Microstructure characteristics of unirradiated LT21 aluminum alloy.

Figure 8 shows the microstructure of unirradiated LT21 aluminum alloy. The second phase particles were existed in the matrix, which are elongated and rounded. The rod-like second phase particles are about 500 nm in diameter and more than 1300 nm in length. EDS analysis showed that the second phase mainly contained Mg and Si elements.

**Figure 9:**
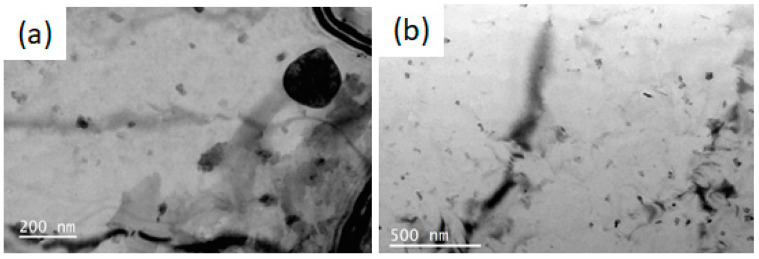
Microstructure characteristics of irradiated LT21: (**a**) thermal neutron fluence of 0.54 × 10^21^ n/cm^2^; (**b**) thermal neutron fluence of 2.38 × 10^22^ n/cm^2^.

Figure 9 shows the microstructure of LT21 aluminum alloy after neutron irradiation. The original round particles, which are similar in shape and size to those of unirradiated LT21, can still be seen in the irradiated LT21 aluminum alloy. A certain amount of nanophase structures—nano particles (NP) for which the size is less than 50 nm—were precipitated in the matrix after neutron irradiation. Additionally, as the neutron fluence increases, the density of the nanometer-precipitated phase increases. This phenomenon is similar to other irradiated aluminum alloys [4,14]. These nanophases were produced due to the (*n*, γ) reaction between the aluminum and thermal neutron, resulting in the transmutation of Al into Si through the (*n*, γ) reactions. The solid transmutation product Si can have a strong effect on radiation damage structure. The transmutation Si can be precipitated either by elemental precipitation or by forming Mg_2_Si until the Mg in solid solution is completely consumed in the alloy [4].

## 4. Analysis

### 4.1. Tensile Property Evaluation

**Figure 10:**
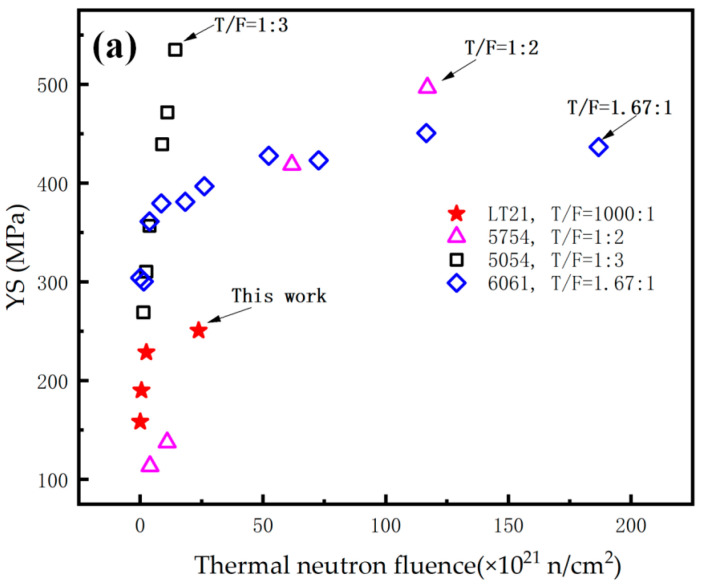

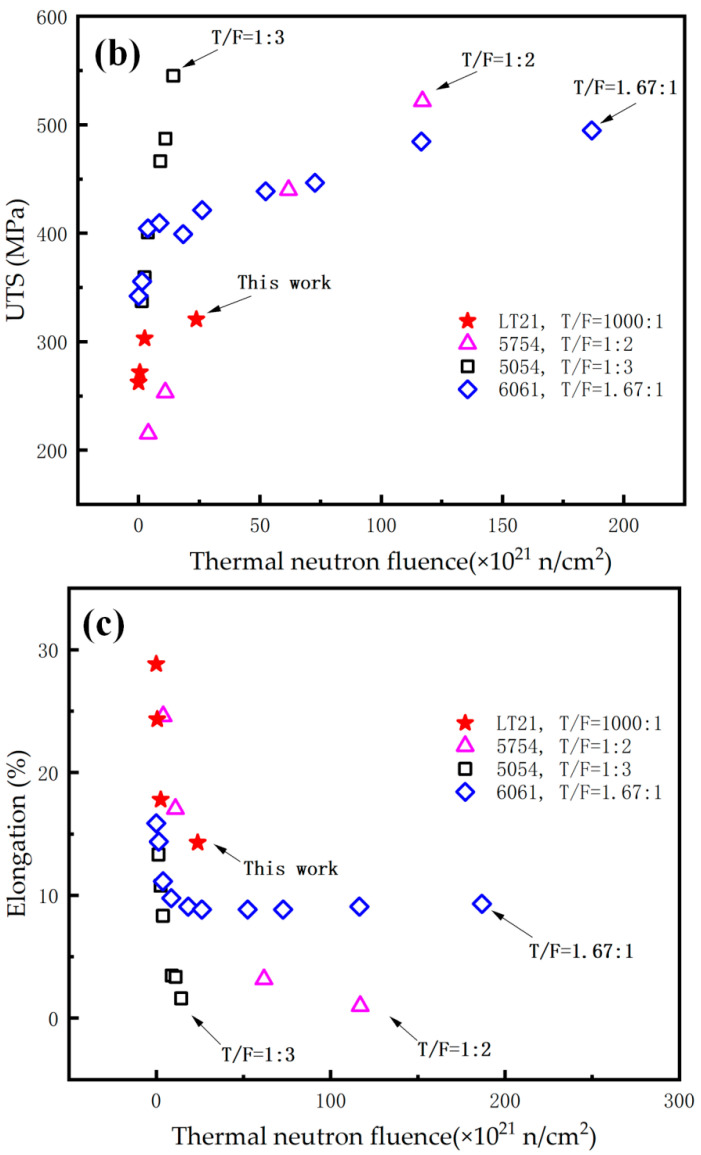
The relationship between (**a**) YS, (**b**) UTS, (**c**) elongation and thermal neutron fluence.

Figure 10a shows the relationship between the YS of aluminum alloy and thermal neutron fluence. The figure shows the test data of this paper and the other data of reference [14,15,16]. The YS of aluminum alloy increased with the thermal neutron fluence increase. However, the increased rates are different among different aluminum alloy because of their different manufacturing process and the ratio of thermal-to-fast flux (TFR). There is no obvious relationship between TFR and the variation trend of YS, which indirectly reflects that the thermal neutron irradiation dominates the irradiation hardening of aluminum alloy. The variation trend of the YS of LT21 aluminum alloy with irradiation fluence is similar to that of other aluminum alloys. The YS of LT21 at 23.8 × 10^21^ n/cm^2^, by contrast, is lower than the other aluminum alloy. This may be related to the fact that LT21 has a TFR of 1000, which is much larger than the other aluminum alloys.

Figure 10b shows the relationship between the UTS of aluminum alloy and thermal neutron fluence. The UTS of aluminum alloy increased with the thermal neutron irradiation fluence increase. However, the increased rates are different among different aluminum alloy because of their different manufacturing process and TFR. There is no obvious relationship between TFR and the variation trend of UTS, which indirectly reflects that thermal neutron irradiation dominates the irradiation strengthening of aluminum alloy. The variation trend of the UTS of LT21 aluminum alloy with irradiation fluence is similar to that of other aluminum alloys.

Figure 10c shows the relationship between the elongation of aluminum alloy and thermal neutron fluence. The elongation of aluminum alloy decreased with the thermal neutron irradiation fluence increase. However, the increased rates are different among different aluminum alloys because of their different manufacturing process and TFR. The variation trend of the elongation of LT21 aluminum alloy with irradiation fluence is similar to that of other aluminum alloys. The elongation of LT21 at 23.8 × 10^21^ n/cm^2^, by contrast, is larger than the other aluminum alloys. This may be related to the fact that LT21 has a TFR of 1000, which is much larger than that of other aluminum alloys, and additionally, the gage length of the specimens is different among different tests.

In summary, the variation trend of YS, UTS, and elongation of LT21 aluminum alloy with the thermal neutron fluence is very similar to that of other aluminum alloys. However, after irradiation, the strength of LT21 is lower than that of other alloys, and the elongation is higher than that of other alloys, especially at the thermal neutron fluence of 23.8 × 10^21^ n/cm^2^. Additionally, the tensile property difference between LT21 and other Aluminum alloys after irradiation may be related to the TFR and tensile test method.

### 4.2. Irradiation Effect Mechanism

According to the results of mechanical analysis and microscopic analysis, it can be inferred that the effect of neutron irradiation on the tensile property of LT21 aluminum alloy is mainly due to the transmutation-produced Si by thermal neutrons. Similar phenomena have been reported by previous studies [3,4,5,16]. The mechanism is speculated to be as follows [3,4]. After neutron irradiation, the transmutation product Si forms in the Aluminum alloy, as the solubility of Si in the Al matrix below 373 K is negligible [4]. Hence, the transmutation-produced Si either precipitates in elemental form or forms Mg_2_Si precipitates in aluminum alloys. The structure, size, and distribution of these precipitates (Si and Mg_2_Si) in the microstructure determines the resulting mechanical properties of the irradiated alloys [4]. For a given volume fraction of precipitates in the microstructure, the precipitates result in higher strength, but lower ductility, and thus cause irradiation hardening. The mechanism continues until saturation in the density of precipitates is expected to occur, leading to the formation of no new Mg_2_Si precipitates. With further irradiation, the hardening continues at a decreasing rate as Mg_2_Si precipitates continue to grow until all the Mg is pulled out from the Al solid solution and leads to a slow irradiation hardening rate. The above analysis shows that transmutation to Si is the main cause of the irradiation effect mechanism of LT21, and this is the same as previous studies on other aluminum alloys [3,4,5,9,14,15,16].

## 5. Conclusions

In this paper, a tensile specimen of LT21 was prepared from the irradiation channel decommissioned by HWRR. By comparing the experimental results of irradiated and nonirradiated LT21 samples, the effect of neutron irradiation on the tensile properties of LT21 aluminum alloy was discussed. The failure mechanism of LT21 aluminum alloy before and after irradiation was analyzed. The conclusions are as follows:

1. Irradiation hardening and irradiation embrittlement occurred on the LT21 after neutron irradiation. With the thermal neutron flux increasing to 2.38 × 10^22^ n/cm^2^, the YS gradually increased from the initial 158 MPa to 251 MPa, the UTS increased from 262 MPa to 321 MPa, and the elongation decreased from 28.8% to about 14.3%.
2. The variation trend of YS, UTS, and Elongation of LT21 with thermal neutron fluence is very similar to that of other aluminum alloys. The neutron irradiation effect on aluminum alloy mainly depends on the thermal neutron, and the specific tensile properties are different among different irradiated aluminum alloys because of their different manufacturing process and TFR, especially at high thermal neutron fluence, e.g., 2.38 × 10^22^ n/cm^2^.
3. A brittle fracture appeared on the LT21 specimen after irradiation, and the proportion of brittle fracture increased with the neutron fluence increased. After neutron irradiation, the LT21 aluminum alloy had been embrittled, but the toughness remained at the neutron irradiation fluence of 2.38 × 10^22^ n/cm^2^.
4. Transmutation to Si is presumed to be the main cause of the radiation effect mechanism of LT21.The nanophase structures, for which the size was less than 50 nm, were precipitated in the LT21 aluminum alloy after neutron irradiation. These nanophases were produced due to the (*n*, γ) reaction of the aluminum element and the thermal neutron, and can cause an increase in strength and a decrease in ductility.

## Figures and Tables

**Table 1:** Chemical composition of A508-3 steel in wt%.

Element	Mg	Si	Fe	Cu	Al
Content	0.96	0.76	0.2	<0.01	Bal.

## Data Availability

Data available on request from the authors.
